# Highly Sensitive Amperometric Detection of Hydrogen Peroxide in Saliva Based on N-Doped Graphene Nanoribbons and MnO_2_ Modified Carbon Paste Electrodes

**DOI:** 10.3390/s21248301

**Published:** 2021-12-11

**Authors:** Ema Gričar, Kurt Kalcher, Boštjan Genorio, Mitja Kolar

**Affiliations:** 1 Department of Chemistry and Biochemistry, Faculty of Chemistry and Chemical Technology, University of Ljubljana, Večna pot 113, 1000 Ljubljana, Slovenia; ema.gricar@fkkt.uni-lj.si; 2 Department of Analytical Chemistry, Insistute of Chemistry, University of Graz, Universitätsplatz 1, 8020 Graz, Austria; kurt.kalcher@uni-graz.at; 3 Department of Chemical Engineering and Technical Safety, Faculty of Chemistry and Chemical Technology, University of Ljubljana, Večna pot 113, 1000 Ljubljana, Slovenia

**Keywords:** electrochemical sensor, hydrogen peroxide, graphene, graphene nanoribbons, amperometry

## Abstract

Four different graphene-based nanomaterials (htGO, N-htGO, htGONR, and N-htGONR) were synthesized, characterized, and used as a modifier of carbon paste electrode (CPE) in order to produce a reliable, precise, and highly sensitive non-enzymatic amperometric hydrogen peroxide sensor for complex matrices. CPE, with their robustness, reliability, and ease of modification, present a convenient starting point for the development of new sensors. Modification of CPE was optimized by systematically changing the type and concentration of materials in the modifier and studying the prepared electrode surface by cyclic voltammetry. N-htGONR in combination with manganese dioxide (1:1 ratio) proved to be the most appropriate material for detection of hydrogen peroxide in pharmaceutical and saliva matrices. The developed sensor exhibited a wide linear range (1.0–300 µM) and an excellent limit of detection (0.08 µM) and reproducibility, as well as high sensitivity and stability. The sensor was successfully applied to real sample analysis, where the recovery values for a commercially obtained pharmaceutical product were between 94.3% and 98.0%. Saliva samples of a user of the pharmaceutical product were also successfully analyzed.

## 1. Introduction

Hydrogen peroxide (H_2_O_2_) is a byproduct in numerous enzymatic processes and in energy conversion devices (fuel cells and Li-air batteries) that utilize the oxygen reduction reaction (ORR). It is widely used as an antimicrobial agent in hygiene and disinfection products, as well as a preservative in food, making it an important analyte in analytical chemistry. Therefore, accurate and precise determination of H_2_O_2_ using rapid, reliable, and cost-effective approaches is of great importance. Many techniques have been developed for H_2_O_2_ determination, such as chromatography [1,2,3], chemiluminescence [4], colorimetry [5,6], and titrimetric analysis [7]. However, the above methods are low-throughput and are often considered complex, expensive, and time consuming. In recent years, many electrochemical approaches have been developed to determine low concentrations of H_2_O_2_ in a high-throughput fashion–in fast, simple, reliable, and inexpensive ways [8,9]. Electrochemical sensors are generally suitable for various matrices, do not require many sample preparation steps, and exhibit high sensitivity and wide concentration range, as well as a low limit of detection (LOD). Both enzymatic [10,11] and non-enzymatic [12,13] sensors have been developed in order to detect H_2_O_2_. While enzymatic approaches do exhibit excellent selectivity and sensitivity, they usually lack stability. Further, the enzyme immobilization process is usually complex and expensive, and the enzyme activity is highly dependent on experimental conditions, such as temperature and pH. Therefore, the development of non-enzymatic electrochemical H_2_O_2_ sensors is of great interest. 

Carbon paste electrodes (CPE) are often used as electrode materials in the development of new sensing platforms because they are robust and can be easily and reproducibly fabricated, have low residual currents in a wide potential window, and offer many possibilities for modification [14]. Various nanomaterials have been used to modify CPE for electrocatalytic H_2_O_2_ detection. Prussian blue, for example, shows high catalytic activity towards H_2_O_2_ [15,16]. However, these electrodes exhibit poor stability and their performance is highly pH dependent [17]. Metallic catalysts, Pt, Cu or Ag, have also been used to determine H_2_O_2_ either by oxidation or reduction. They show high activity towards H_2_O_2_, as well as improved stability compared to Prussian blue electrodes, but their application is limited due to their relatively high prices [18,19,20]. Metal oxides, such as FeO, CuO, Fe_3_O_4_, and MnO_2_ proved to be sufficient alternatives since they are thermally and chemically stable, abundant in nature, and environmentally friendly. In particular, manganese oxides have been found to be the most appropriate materials to apply for H_2_O_2_ sensing because of their outstanding catalytic activity towards the decomposition of H_2_O_2_ [19,20,21,22]. Recently, non-metallic nanomaterials, especially graphene-based, have attracted interest in sensing applications due to their superior properties. H_2_O_2_ is often used in substantial concentrations in oral hygiene (up to approximately 3%) and teeth whitening (up to 10%) products [23]. Since H_2_O_2_ is known to cause toxicity at the site of contact as well as to form hydroxyl radicals, whose activity can lead to DNA damage and cell death [24], it is important to assess whether the use of oral hygiene and teeth whitening products is a cause for concern. In this work, an over-the-counter oral hygiene product was analyzed. Additionally, residual content of H_2_O_2_ in saliva after the use of the hygiene product was determined.

Graphene, a two-dimensional monoatomic layer of carbon atoms, has been widely used in electrochemistry due to its large surface area, mechanical stiffness, high thermal conductivity, biocompatibility, ease of functionalization, wide potential window, and fast electron transport [25]. Various combinations of graphene (and other carbon nanomaterials) with metal oxides have been reported to positively influence sensing of different analytes. Graphene nanoribbons (GNR), essentially narrow strips of graphene, are quasi-1D carbon allotrope, which are believed to have numerous advantages over graphene due to their peculiar electronic structure and morphology (high aspect ratio compared to 2D materials), such as band gap opening and higher chemical and electrochemical reactivity [26]. Doping of graphene materials with nitrogen is reported to further enhance the electrochemical activity, adsorption and activation of analytes, promote charge transfer, and facilitate further modifications when used in electrochemical sensors [25,27].

The number of works that report the use of quasi-1D graphene-based nanomaterials in electrochemical sensors is increasing, however, GNR are still poorly researched in the H_2_O_2_ sensor development. This work is one of the few reported [12,28,29] that use this type of material for sensor development. According to the extensive literature review, it is the first work that uses N-doped GNR in combination with MnO_2_ to develop a sensor for trace concentrations of H_2_O_2_. The authors claim that the sensor described below has such a low LOD and high sensitivity precisely because of the use of N-doped GNR. The main objective of this work was to develop a highly sensitive, simple, and reliable non-enzymatic H_2_O_2_ sensor using the optimal combination of synthesized graphene-based nanomaterials and MnO_2_. Modified CPE were tested and optimized to obtain a sensor with desirable properties, and the first steps towards real sample analysis were taken.

## 2. Materials and Methods

### 2.1. Chemicals and Solutions

Buffer solutions were prepared by weighing Na_2_HPO_4_ and NaH_2_PO_4_ (both Sigma-Aldrich, Darmstadt, Germany) in the appropriate ratio and dissolving them in ultrapure water with resistivity of >18.2 MΩ/cm (Millipore/MilliQ system; MQ) to obtain a 0.1 M buffer solution with a pH of 7.41. Thus prepared, phosphate buffer (PB) was used as a medium for all other solutions. All chemicals used for solutions were obtained commercially and were of analytical reagent grade. H_2_O_2_ solutions used for measurements were prepared daily by diluting the commercially obtained 30 wt % H_2_O_2_ (Sigma-Aldrich, Darmstadt, Germany), which was also standardized with KMnO_4_ titration prior to use.

### 2.2. Synthesis of Graphene-Based Nanomaterials

Heat-treated graphene oxide (htGO) and nitrogen-doped heat-treated graphene oxide (N-htGO) were prepared from graphite powder (Timrex KS6L, Tuscon, AZ, USA), while heat-treated graphene oxide nanoribbons (htGONR) and nitrogen-doped heat-treated graphene oxide (N-htGONR) were prepared from multi-walled carbon nanotubes (MWCNTs) (M-grade MWCNTs, NanoTechLabs, Yadkinville, NC, USA). H_2_SO_4_ (ACS reagent, 95.0–98.0%, Sigma-Aldrich, Darmstadt, Germany), H_3_PO_4_ (ACS reagent, ≥85 wt % in H_2_O, Sigma-Aldrich, Darmstadt, Germany), KMnO_4_ (ACS reagent ≥ 99%, Sigma-Aldrich, Darmstadt, Germany), HCl (ACS reagent, 37%, Sigma-Aldrich, Darmstadt, Germany) and H_2_O_2_ (ACS reagent, 30 wt %, Sigma-Aldrich, Darmstadt, Germany) were all used as reagents in the synthesis of graphene-based nanomaterials. Firstly, graphene oxide (GO) and graphene oxide nanoribbons (GONR) were prepared according to an improved Hummer’s method [30] using graphite or MWCNTs (20 g), concentrated H_2_SO_4_ and H_3_PO_4_ in 9:1 volume ratio and six aliquots of 20 g KMnO_4_ to oxidize the starting materials. After 10 days of continuous stirring, the reaction was quenched with 800 mL ice and 15 mL 30 vol % H_2_O_2_. The reaction mixture was centrifuged and thoroughly washed with 10 % HCl and MQ, then freeze-dried. Secondly, GO and GONR were heat-treated in (a) N_2_ atmosphere (30 mL/min flow) to obtain htGO and htGONR, (b) in NH_3_ atmosphere (30 mL/min flow) to obtain N-htGO and N-htGONR. The synthesized materials were characterized by scanning electron microscopy (SEM) using a field emission electron microscope Zeiss ULTRA plus SEM (Cazl Zeiss NTS Ltd., Oberkochen, Germany), Brunauer–Emmett–Teller (BET) analysis using an ASAP 2020 Micrometrics instrument (Micrometrics, Norcross, GA, USA), X-ray photoelectron spectroscopy (XPS) using a PHI Quantera SXM photoelectron spectrometer analyzer (PHI, Chanhassen, MN, USA), C, H, N analysis using PerkinElmer CHN Analyzer 2400 II (PerkinElmer, Rodgau, Germany), Raman spectroscopy using Raman/AFM WITec Alpha 300RAS instrument (WITec, Ulm, Germany), and ICP–MS using an ICP–MS Agilent Technologies 7900 instrument (Agilent Technologies Ltd., Santa Clara, CA, USA). For more details regarding XPS and Raman analyses please see Supporting Information pg. S1–S2. The syntheses and characterization procedures are described in more detail in our previous work [31].

### 2.3. Preparation of the Electrode Surface

Carbon pastes were prepared from graphite powder (synthetic; <20 µm; Sigma-Aldrich, Germany) and silicone oil AP 100 (Sigma-Aldrich, Darmstadt, Germany): 1.0 g of graphite powder was thoroughly mixed with 950 µL silicone oil in the agate mortar until a homogeneous paste was obtained and stored in a sealed refrigerated container until use. CPE were prepared by packing the prepared paste into a circular Teflon holder with 8 mm diameter and polishing the surface. 

Synthesized nanomaterials were ultrasonically dispersed in 50% ethanol for 90 min. One milligram/milliliter dispersions of each graphene-based nanomaterial and MnO_2_ (<1 µm; Sigma-Aldrich, Darmstadt, Germany), respectively, were prepared. Appropriate volumes of a chosen graphene-based nanomaterial and MnO_2_ dispersions were mixed together with neutralized Nafion (Perfluorinated resin solution containing Nafion^®^ 1100W; Sigma-Aldrich, Darmstadt, USA) and 5 mg/mL solution of glycerol (Sigma-Aldrich, Darmstadt, Germany). Obtained mixture was again sonicated for 30 min before drop-casting two 10 µL layers of the mixture onto the CPE surface. Different dispersions were prepared by systematically changing the concentrations of each of the four components, one by one, and testing the electrochemical characteristics of the prepared modified CPE. All synthesized graphene-based nanomaterials were investigated for their properties in sensor application.

### 2.4. Electrochemical Measurements

Electrochemical measurements were performed on a Metrohm Autolab potentiostat PGSTAT302N (Metrohm, Herisau, Switzerland) using the three-electrode system, where the working electrode was CPE, the auxiliary electrode was a Pt wire, and the reference electrode was Ag/AgCl electrode (LL ISE 6.0750.100 Ag,AgCl/3 M KCl Metrohm, Herisau, Switzerland). CPE were first characterized by cyclic voltammetry (CV) and electrochemical impedance spectroscopy (EIS). Characterization of the electrode surface was performed in a 5 mM solution of K_3_[Fe(CN)_6_]/K_4_[Fe(CN)_6_] (both Sigma-Aldrich, Darmstadt, Germany). Depending on the observed parameters, the best CPE were further used for amperometric detection of H_2_O_2_. Before amperometric measurements, the CPE was immersed into the phosphate buffer solution and was conditioned by performing 10 CV cycles in the potential window from –0.2 V to +0.8 V (vs. Ag/AgCl), with 100 mV/s scan rate. Then the amperometric measurements took place, where H_2_O_2_ was added directly into the electrochemical cell and the electric current was recorded at the constant operating potential.

To evaluate the selectivity of the presented sensor, its response to seven potential biological interferences was investigated. Uric acid (Sigma-Aldrich, Darmstadt, Germany), ascorbic acid (Sigma-Aldrich, Darmstadt, Germany), dopamine (Sigma-Aldrich, Darmstadt, Germany), xanthine (MilliporeSigma, Burlington, MA USA), paracetamol (Sigma-Aldrich, Darmstadt, Germany), BSA (Sigma-Aldrich, Darmstadt, Germany), and glucose (Sigma-Aldrich, Darmstadt, Germany) were all obtained commercially. The procedure was quite similar to the amperometric detection of H_2_O_2_–standard solution of H_2_O_2_ was added to the continuously stirred buffer solution followed by a specified amount of the potential interference. The current change was calculated and compared to the current change caused by H_2_O_2_.

Pharmaceutical sample Oroxid (obtained commercially at Lekarna Ljubljana, Slovenia) was sequentially diluted before the addition into the electrochemical cell with no other sample preparation steps. Saliva samples collected before, immediately after the use of Oroxid, and after rinsing of the mouth with water were filtered and added to the electrochemical cell without any additional sample preparation steps, respectively. In order to analyze the saliva samples, the user strictly followed the instructions on the bottle of the product: spraying the solution into the mouth with 4 pressures of the spray, gargling for 30 s and spitting it out.

## 3. Results and Discussion

### 3.1. Synthesis and Characterization of Graphene-Based Nanomaterials

**Figure 1:**
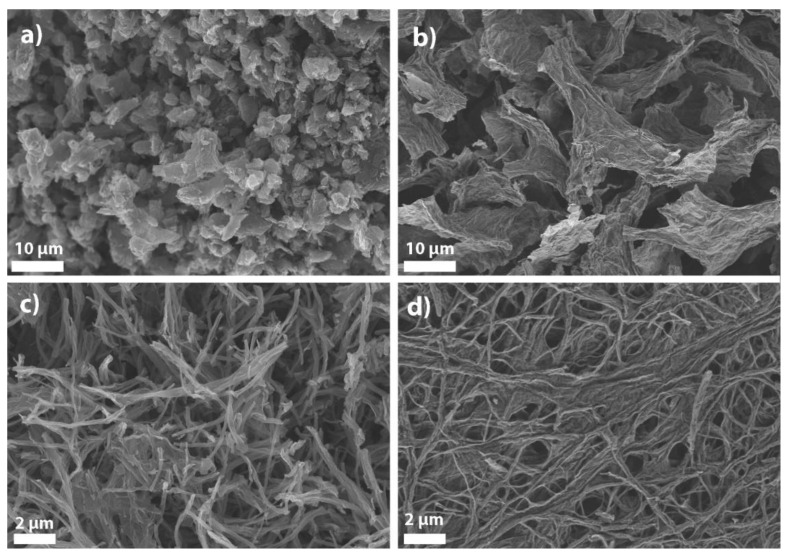
SEM images of (**a**) htGO (KS6L), (**b**) N-doped htGO (KS6L), (**c**) htGONR (M-grade), and (**d**) N-doped htGONR (M-grade) materials.

Graphene-based nanomaterials were synthesized according to a two-step top-down approach. Quasi-1D materials were synthesized by oxidative longitudinal unzipping of MWCNTs, while 2D materials were prepared by oxidative chemical exfoliation of graphite flakes. The formation of sheets (GO) and ribbons (GONR) after this synthesis step were confirmed morphologically using SEM imaging (see Figure S1). The second synthesis step was pyrolysis under reducing NH_3_ or inert N_2_ atmospheres. All pyrolyzed materials were also morphologically analyzed by SEM (Figure 1). The images clearly show exfoliated materials with relatively high specific surface area. htGO and N-doped htGO (Figure 1a,b) show crumpled flake-like materials, while htGONR and N-doped htGONR (Figure 1c,d) show quasi 1D ribbon-like materials. During the pyrolysis under N_2_ atmosphere, oxygen functional groups are reduced or decomposed, additional defects are introduced into the structure, and the material is furtherly exfoliated [32]. During the pyrolysis under NH_3_ atmosphere, N-functional groups are introduced into the structure in addition to the above-described processes. All processes significantly influence the properties of graphene-based nanomaterials. The detailed characterization of all the materials was published in our previous work [31] and is summarized below for the purpose of understanding the improved sensing properties.

Surface area is an important property of materials used in sensor development. The BET analysis was employed to examine the specific surface area of the synthesized materials. The results (Table 1) show a significant increase in surface area for all materials compared to natural graphite (0.6 m^2^/g [33]), which was used to prepare CPE. Metal impurities were also investigated by ICP-MS as they can strongly affect the electrochemical properties of the material. In this case, Mn-impurities were of particular interest since MnO_2_ was used to catalyze H_2_O_2_ reaction on the electrode. The results of the ICP-MS analysis show a content between 1.2 and 4.1 mg/g of Mn-impurities in all materials. The Mn-impurities are residues of the reagent that was used in the first step of the synthesis of GO. The degree of reduction and N-doping during pyrolysis was investigated by C, H, N analysis. The concentration of nitrogen is 1 at % higher in the case of 2D material than in the case of quasi-1D material. An important parameter is also the incidence of pyridinic N-groups in the doped materials, since they are considered to be highly reactive and the main cause of enhancement of electrocatalytic activity [34]. XPS analysis was carried out to investigate the distribution of N-configuration. Pyridinic-N, pyrrolic-N, graphitic-N, and oxidized-N configurations were of interest. Deconvolution of N1s XPS core-level spectra shows 41.5 and 44.2 at % pyridinic-N and 23.4 and 19.7 at % pyrrolic-N content in N-htGONR and N-htGO. This indicates higher electrocatalytic activity of N-doped materials in comparison to non-doped materials due to the higher concentration of pyridinic-N. Furthermore, pyridinic and pyrrolic functional groups are known to strongly bind metals and by that improve the overall stability of the material [35]. Since the reactivity of the material is also dependent on the concentration of defects in the structure, Raman spectra were examined. Observing D and G peaks, it was estimated that all materials exhibit high defect density.

Taking into account all the data obtained from material characterization, one can assume that all four graphene-based nanomaterials will improve the electrochemical characteristics of the CPE surface due to the increase in specific surface area, higher conductivity and activity towards H_2_O_2_ due to metal impurities, higher reactivity due to high concentration of defects and in case of N-doped materials, pyridinic and pyrrolic functional groups. N-doped materials also offer enhanced charge transfer characteristics [25], while quasi-1D materials exhibit a higher ratio of edge to basal carbons than 2D materials, which increases their reactivity. It is expected that N-doped and quasi-1D materials will exhibit higher reactivity towards H_2_O_2_ decomposition, similarly to the reported higher activity towards oxygen reduction reaction, described by Nosan et al. [31].

### 3.2. Electrode Preparation, Characterization, and Optimization

As reported in previous studies [19,36,37,38], the modified CPE exhibit many desirable analytical parameters, such as low LOD, adequate sensitivity, durability, and reproducibility. Moreover, the simplicity and many possible modification mechanisms (e.g., drop casting [19] and bulk modification [39,40,41]) make CPE a convenient starting point for the development of electrochemical sensors. In this work, CPE were modified by drop casting a dispersion of a graphene-based nanomaterial (either htGO, N-htGO, htGONR, or N-htGONR), MnO_2_, and Nafion onto the electrode surface. Each component of the dispersion was expected to serve its purpose–MnO_2_ is known to have an outstanding activity towards H_2_O_2_; graphene materials exhibit a large surface area which influences the sensitivity of the sensor, enables good particle distribution of the MnO_2_, promotes charge transfer, and improves the electronic conductivity of the electrode; Nafion serves as a binder that stabilizes the layer of MnO_2_ and graphene while maintaining high ionic conductivity of the electrode surface. The composition of the dispersion was systematically changed to obtain a CPE with optimal parameters.

Starting concentrations of both graphene-based nanomaterials and MnO_2_ in the dispersion were 0.4 mg/mL, respectively, obtained by mixing the initial separate 1.0 mg/mL dispersions. When the ratio was changed to be in favor of MnO_2_, the conductivity of the electrode surface significantly decreased, while no significant change was observed when the ratio was changed to be in favor of graphene-based nanomaterial. The ratio remained at 1:1 for the rest of the study. When higher concentrations (0.8, 1.0 mg/mL) of both materials were used, the dispersion was not homogeneous even upon visual inspection and the layers cast on the electrode were unstable when immersed in a solution–the particles started to crumble off the surface during the measurements. To increase the number of nanoparticles while maintaining a stable and repeatable electrode surface, two layers of the optimal dispersion were used.

**Figure 2:**
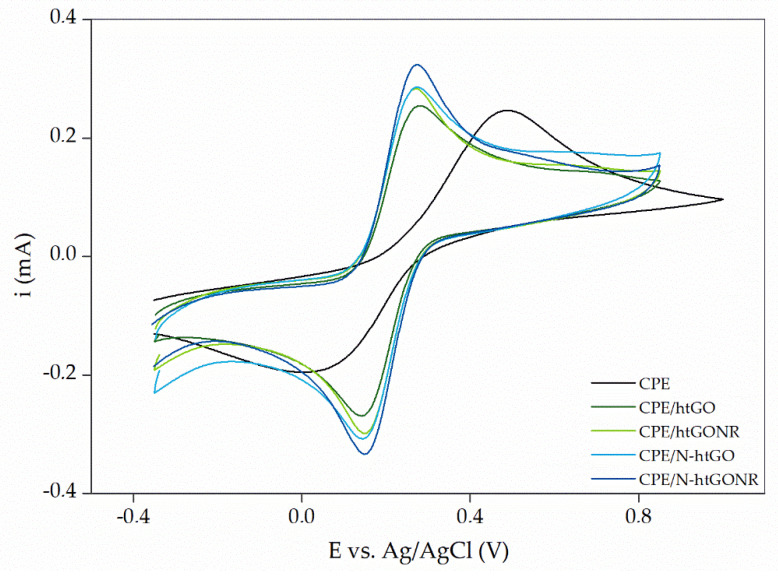
Cyclic voltammograms for measurements with bare CPE, CPE/htGO/MnO_2_/Nafion, CPE/N-htGO/MnO_2_/Nafion, CPE/htGONR/MnO_2_/Nafion, and CPE/N-htGONR/MnO_2_/Nafion in 0.1 M PB solution (pH 7.4) containing 5 mM [Fe(CN)_6_]^3–/4–^.

After drop casting and drying, the modified CPE were immersed in 0.1 M PB (pH 7.4), and their electrochemical properties were examined using CV and EIS. Both analyses were performed in the 0.1 M PB solution (pH 7.4) containing 5 mM [Fe(CN)_6_]^3–/4–^. First, it was determined which of the four tested graphene-based nanomaterials exhibited the optimal electrochemical properties in combination with other components of the electrode. Figure 2 shows cyclic voltammograms for bare CPE, CPE/htGO/MnO_2_/Nafion, CPE/N-htGO/MnO_2_/Nafion, CPE/htGONR/MnO_2_/Nafion, and CPE/N-htGONR/MnO_2_/Nafion. It is clearly seen that the combination of materials used for electrode modification, regardless of the choice of the graphene-based nanomaterial, improves the properties of the CPE. The redox peaks for bare CPE are significantly lower and wider than those of the modified electrodes. The peak-to-peak separation value (ΔE_pp_) is also an important parameter that provides information about the electron transfer rate at the electrode. The ideal ΔE_pp_ value is 59.2 mV, as shown by the Nernst equation [42]. The ΔE_pp_ values are 492, 128, 141, 125, and 116 mV for bare CPE, CPE/htGO/MnO_2_/Nafion, CPE/N-htGO/MnO_2_/Nafion, CPE/htGONR/MnO_2_/Nafion, and CPE/N-htGONR/MnO_2_/Nafion, respectively. These results indicate that the electron transfer rate is highest in the case of using N-htGONR, and therefore N-htGONR is the optimal material of choice. This hypothesis is supported by the peak heights in Figure 2 and is also consistent with the above discussed edge effects of the quasi-one-dimensional materials and the incorporated nitrogen functional groups.

**Figure 3:**
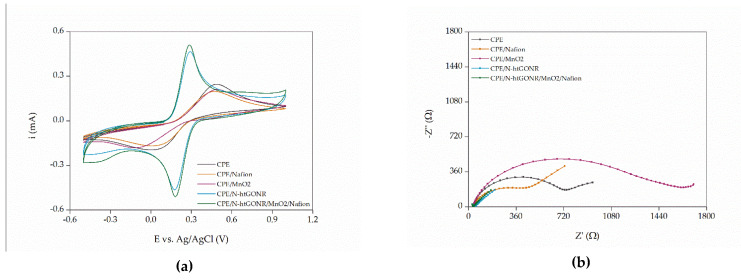
Cyclic voltammograms (**a**) and Nyquist plots (**b**) for measurements with bare CPE, CPE/Nafion, CPE/N-htGONR, CPE/MnO_2_, and CPE/N-htGONR/MnO_2_/Nafion in 0.1 M PB solution (pH 7.4) containing 5 mM [Fe(CN)_6_]^3–/4–^.

To furtherly confirm the choice of materials, a similar study was performed with the bare CPE, CPE/Nafion, CPE/MnO_2_, CPE/N-htGONR, and CPE/N-htGONR/MnO_2_/Nafion. The cyclic voltammograms in Figure 3a show significantly lower ΔE_pp_ (about 100 mV compared to 320 mV for the bare CPE) and higher redox peaks whenever N-htGONR were used to modify the CPE, indicating that N-htGONR do in fact promote the charge transfer process. Slightly higher peaks were observed when N-htGONR and MnO_2_ were used, suggesting a synergistic effect of joint dispersion on the electrochemical properties. Wu et al. [28] described a similar phenomenon for a composite material of reduced graphene oxide nanoribbons and MnO_2_. The conductivity of the electrode surface is important for the analytical performance of the sensor, since it influences the sensitivity, as well as the reliability of the response. Therefore, Nyquist plots were recorded (Figure 3b) and charge transfer resistance (R_ct_) was determined. Obviously, the desired R_ct_ value was as low as possible. The R_ct_ values for CPE, CPE/Nafion, CPE/N-htGONR, CPE/MnO_2_, and CPE/N-htGONR/MnO_2_/Nafion were 725 Ω, 422 Ω, 1584 Ω, 19 Ω, and 6 Ω, respectively. As expected, the results were in agreement to those of CV measurements. The superior electrochemical properties of CPE/N-htGONR/MnO_2_/Nafion could be assigned to the high surface area and high conductivity provided by N-htGONR particles, as well as the above suggested synergistic effect between MnO_2_ and graphene nanomaterials.

Amperometry was chosen for the detection of H_2_O_2_, because it is a very simple, rapid and efficient method for the detection of target analyte and is also the method of choice in many previously published works on H_2_O_2_ detection [13,28]. The operating potential was chosen by recording a CV in a 1 mM solution of H_2_O_2_ in 0.1 M PB (pH 7.4) and observing the oxidation peak. Potentials between 0.50 and 0.75 V were tested by recording amperograms, where four consecutive additions of 50 µM H_2_O_2_ were added, and the current change was observed. Finally, 0.65 V was chosen as the optimal operating potential as it exhibited the highest current response.

### 3.3. Linear Range, Sensitivity, Durability, Reproducibility, and Selectivity Studies

**Figure 4:**
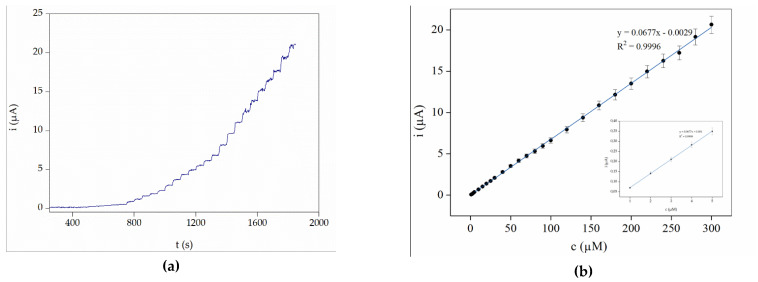
(**a**) Amperogram and (**b**) calibration curve obtained by adding five times 1 µM, five times 5 µM, seven times 10 µM and ten times 20 µM aliquots of H_2_O_2_ standard solution (in 0.1 M PB with pH 7.4). Operating potential was 0.65 V. The insert of Figure 4 (**b**) shows five additions of 1 µM aliquots, used to calculate LOD.

The analytical performance of the CPE/N-htGONR/MnO_2_/Nafion sensor was investigated in the next step. The amperometric response for a known amount of H_2_O_2_ in a stirred 0.1 M PB (pH 7.4) was measured at 0.65 V. Successive additions of a standard H_2_O_2_ solution were added to the solution. The calibration curve obtained by this method showed a wide linear range from 1.0 to 300 µM. Examples of measured amperograms and their corresponding calibration curves are shown in Figure 4a,b, respectively. The sensitivity of the proposed sensor is satisfactory at 0.135 µAµM^–1^cm^−2^. LOD was calculated according to the 3S/k method [43] and it was found to be 0.08 µM, which is one of the lowest LOD values found in the literature for similar sensing systems (Table 2).

**Figure 5:**
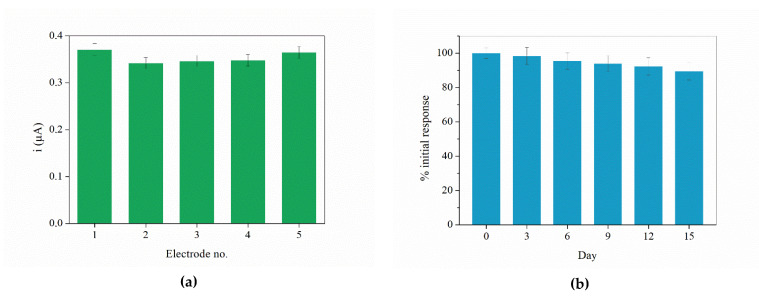
(**a**) Average current change for 5 µM H_2_O_2_ additions for each of the prepared electrodes in reproducibility study. (**b**) Percentage of the sensor’s retained initial response after 0–15 days in durability study.

The lifetime of the proposed sensor was evaluated at an interval of 21 days. Measurements were performed every three days. The electrode was stored at room temperature in a dark place between measurements. As shown in Figure 5b, the sensor retained 89.6% of its initial response after 15 days, confirming its durability. The response after 18 and 21 days drops to 88.5% and 84.8% of the initial response, respectively, indicating that the electrode should not be used for more than two weeks in order to obtain optimal results. The reproducibility was studied by performing amperometric measurements in 0.1 M PB (pH 7.4) with five 5 µM H_2_O_2_ additions. Five individual electrodes, prepared on different days using the same procedure, were used for reproducibility study. The average current change for each prepared electrode is shown in Figure 5a. The calculated RSD value was 3.5%. According to these results it can be concluded that the sensor exhibits good sensitivity and durability, as well as LOD.

The selectivity of the sensor was investigated by analyzing the response of the sensor in the presence of seven interfering species commonly found in biological samples: uric acid, ascorbic acid, paracetamol, dopamine, xanthine, bovine serum albumin, and glucose. The response was measured by consecutively adding 10 µM H_2_O_2_ standard solution three times and an aliquot of an interfering species of the same concentration. This procedure was repeated three times for each studied interference. It was concluded that uric acid, xanthine, bovine serum albumin, and glucose did not interfere with the response of the sensor as no current response occurred after their additions, while paracetamol, ascorbic acid, and dopamine interfered significantly. While this study suggests that the sensor might not be suitable for all biological samples, it can be applied to certain studies in food and pharmaceutical analysis. Under certain conditions it can be used for the analysis of less complex biological samples, e.g., saliva, when it can be asserted with certainty that the subject did not ingest any ascorbic acid or paracetamol in the last few hours.

### 3.4. Real Sample Analysis

Teeth whitening products usually contain about 3.5% of H_2_O_2_ [45]. Since H_2_O_2_ is known to form free radicals, including hydroxyl radicals, which have been implicated in various stages of carcinogenesis [46], the concern for potential systemic toxicity of tooth whitening and other oral hygiene products containing H_2_O_2_ is logical. The user of home teeth whitening systems is also in danger of potentially ingesting H_2_O_2_, which leads to gastrointestinal irritation and possible gas embolisms [24]. In a recent study the concentration of H_2_O_2_ was measured in saliva during and after tooth whitening by a colorimetric method and it confirmed that the tooth whitening process is safe [23]. The sensor proposed in this work was also used to test the H_2_O_2_ content in an oral hygiene product, as well as in saliva immediately after the application of the hygiene product under controlled conditions.

The main objectives of this study with real samples were first to confirm that the proposed sensor has a good recovery value and second that it can be used to some extent for the analysis of biological samples, taking into account the results of interference studies. Oroxid, an over-the-counter oral hygiene product, contains nominally 3% H_2_O_2_, as well as flavors, stabilizers, and Actipone^®^ PX3. The pharmaceutical was diluted and analyzed using the proposed sensor. The recovery values ranged from 94.3% to 98.0% as shown in Table 3. A representative amperogram for the analysis of the pharmaceutical sample is shown in Figure S2. For the saliva sample analysis, the subject applied the product according to the instructions on the bottle. To increase reliability of the results, the subject did not eat or drink anything for 2 h prior to the collection of the samples. Saliva samples were collected and analyzed right before and after use of the product. Two representative amperograms of saliva analyses are shown in Figures S3 and S4. Observing the average current after each addition of the saliva sample, it is clear that no reaction takes place at the electrode surface in case of saliva sampled before use and that there is residual H_2_O_2_ still present in the saliva sample after use of the product. The results suggest that the residual content of H_2_O_2_ in the saliva samples after use was between 30 and 40 ppm, while there was no H_2_O_2_ detected in the saliva collected prior to the use of the product, as well as in the saliva collected after thorough rinsing of the mouth. After an extensive literature review it was concluded that this is the first study to report the contents of residual H_2_O_2_ after the use of oral hygiene products in salivary samples using electrochemical sensing. An investigation by Pournaghi-Azar et al. [16], however, revealed the content of H_2_O_2_ during at-home teeth whitening. Considering the nominal H_2_O_2_ contents in teeth whitening product versus oral hygiene products, our results are in very good agreement with those obtained by the referenced study.

## 4. Conclusions

For the development of the proposed H_2_O_2_ sensor, four graphene-based nanomaterials were tested. N-htGONR was found to be the best for this application, firstly due to its quasi-one-dimensional structure and high aspect ratio (edge effect) compared to N-htGO and htGO, and secondly due to the effect of nitrogen functional groups (especially pyridine) compared to non-doped htGONR. N-htGONR was impregnated with MnO_2_ and Nafion and cast onto the CPE surface. CPE were chosen as the fundamental starting point of this research since they are easy to prepare, offer many possibilities for modification, and are robust. The developed sensor was successfully used to determine H_2_O_2_ concentrations by an amperometric method. The linear range of the proposed sensor was between 1.0 and 300 µM, while the LOD was 0.08 µM, which is one of the lowest LOD values found in the literature for a comparable sensor. Sensitivity, durability, reproducibility, and selectivity were also studied and yielded excellent results. The practical application of the sensor was then tested for two real samples. The developed sensing platform offers an excellent starting point for further research and applications, especially since graphene-based nanoribbons are not commonly used for sensing applications. Finally, this work could have a great impact in development of online sensors for various applications including health, food, and energy fields.

## Figures and Tables

**Table 1:** Results of BET analysis, C, H, N analysis, and Mn concentration, obtained by ICP-MS analysis [31].

Material	C (at %)	H (at %)	N (at %)	Pyridinic N (at %)	S_BET_ (m^2^/g)	*w* (Mn) (mg/g)
htGO	76.9	12.7	0.0	0.0	602.1	4.1
htGONR	72.3	20.1	0.0	0.0	296.5	1.6
N-htGO	75.2	9.1	8.2	3.62	129.7	2.9
N-htGONR	73.1	9.2	7.2	2.99	85.4	1.2

**Table 2:** Comparison of previously reported non-enzymatic electrochemical H_2_O_2_ sensors using similar approaches with the proposed CPE/N-htGONR/MnO_2_/Nafion sensor.

Electrode Composition	Linear Range (µM)	Slope (µA/µM)	LOD (µM)	Ref.
CPE/N-htGONR/MnO_2_/Nafion	1.0–300	0.0677	0.08	This work
MnO_2_-MWCNT/CPE	–	0.0018	70.6	[19]
MnO_x_/CNW	40–10230	0.439	0.55	[44]
MnO_2_/rGONR	0.25–2455	0.0142	0.071	[28]
PB/Pd-Al	5–34	0.0508	4	[16]

**Table 3:** Comparison of previously reported non-enzymatic electrochemical H_2_O_2_ sensors using similar approaches with the proposed CPE/N-htGONR/MnO_2_/Nafion sensor.

Measurement No.	Analyte Found (%)	Recovery (%)
1	2.83	94.3
2	2.94	98.0
3	2.88	96.0

## Data Availability

Data sharing not applicable.

## Supplementary Materials

The following are available online at www.mdpi.com/article/10.3390/s21248301/s1: Figure S1: (a) SEM image confirming the exfoliation of graphite flakes and formation of 2D sheet structures. (b) SEM image confirming the unzipping of MWCNT and formation of quasi-1D ribbon structures. Figure S2: Amperogram for the measurement of pharmaceutical sample. Electrochemical cell contained 20 mL 0.1 M PB (pH 7.4); the measurement was carried out at 0.65 V. Five additions of 5 µM standard solution were added, followed by three additions of 100,000-fold diluted pharmaceutical sample. Figure S3: Amperogram for the measurement of saliva sample before the use of oral hygiene product. Electrochemical cell contained 20 mL 0.1 M PB (pH 7.4); the measurement was carried out at 0.65 V. Five additions of 5 µM standard solution were added, followed by three additions of 50 µL of saliva. Figure S4: Amperogram for the measurement of saliva sample after the use of oral hygiene product. Electrochemical cell contained 20 mL 0.1 M PB (pH 7.4); the measurement was carried out at 0.65 V. Five additions of 5 µM standard solution were added, followed by three additions of 50 µL of saliva.
