# Supplementary material for: Highly Sensitive Amperometric Detection of Hydrogen Peroxide in Saliva Based on N-Doped Graphene Nanoribbons and MnO2 Modified Carbon Paste Electrodes
Source: Sensors (Basel). 2021 Dec 11;21(24):8301. doi: 10.3390/s21248301 (PMC8707399; doi:10.3390/s21248301)
Supplement: Supplementary file 1 [file sensors-21-08301-s001.zip › sensors-1499580-supplementary.pdf]

Supplementary information

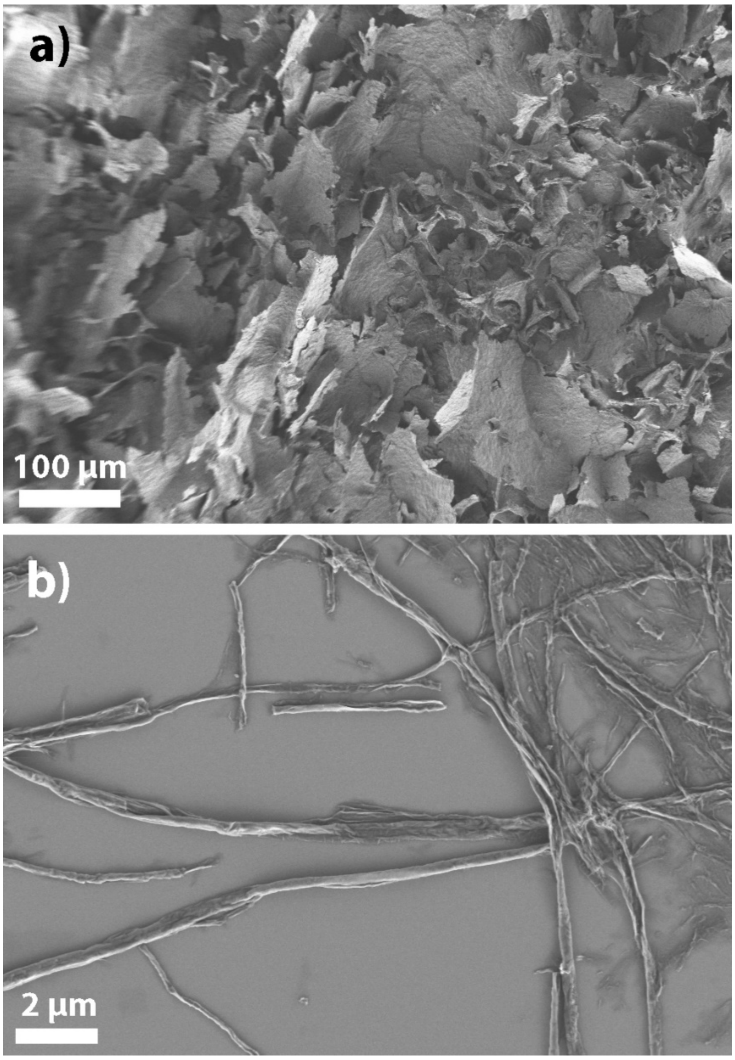

Figure S1: a) SEM image confirming the exfoliation of graphite flakes and formation of 2D sheet structures. b) SEM image confirming the unzipping of MWCNT and formation of quasi-1D ribbon structures.

X-ray Photoelectron Spectroscopy (XPS) Measurements. XPS measurements were performed using a PHI Quantera SXM photoelectron spectrometer analyzer with a monochromatic Al Kα X-ray source. Survey spectra were measured using a pass energy of 280 eV at a resolution of 1 eV/step and a total integration time of 0.2 s/point. Core-level spectra were measured using a pass energy of 112 eV at a resolution of 0.1 eV/step and a total integration time of 0.2 s/point. Deconvolution was performed using CasaXPS software, with a Shirley type background and 70 – 30 % Gaussian–Lorentzian peak shapes for all peaks but N oxide configuration, which was

fitted using an asymmetric peak shape, the constrain 1.2 – 1.6 for FWHM was used. Binding energy was calibrated and corrected for C1s peak at 284 eV.

Raman Spectroscopy. Raman spectroscopy was done on Raman/AFM WITec Alpha 300RAS with green Laser emitting light at 532 nm and 5 mW. The integration time was 10 s.

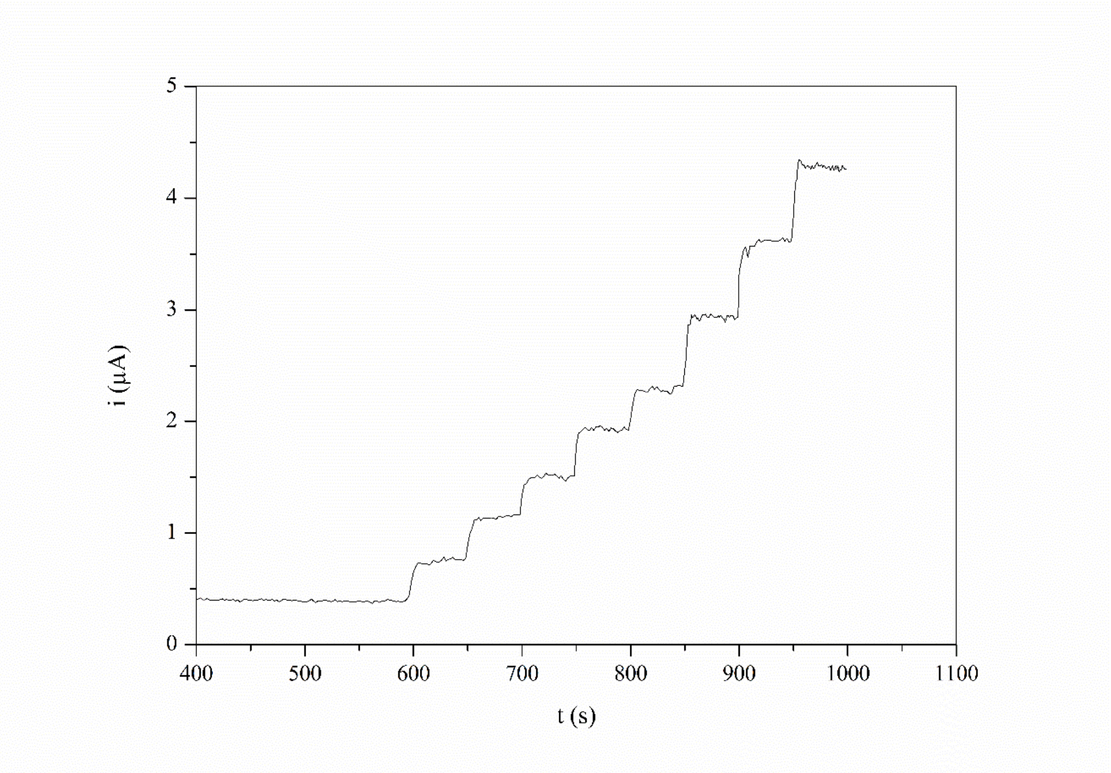

Figure S2: Amperogram for the measurement of pharmaceutical sample. Electrochemical cell contained 20 mL 0.1 M PB (pH 7.4); the measurement was carried out at 0.65 V. Five additions of 5 μM standard solution were added, followed by three additions of 100000-fold diluted pharmaceutical sample.

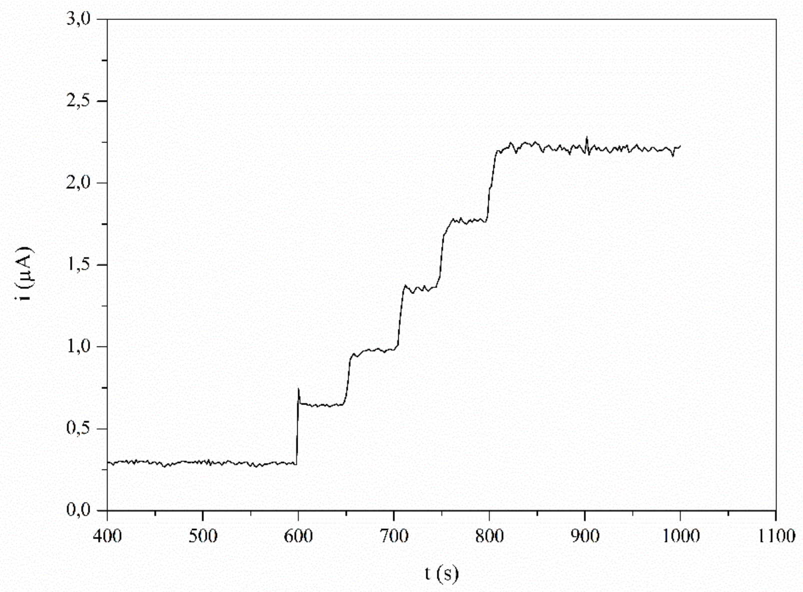

Figure S3: Amperogram for the measurement of saliva sample before the use of oral hygiene product. Electrochemical cell contained 20 mL 0.1 M PB (pH 7.4); the measurement was carried out at 0.65 V. Five additions of 5 µM standard solution were added, followed by three additions of 50 µL of saliva.

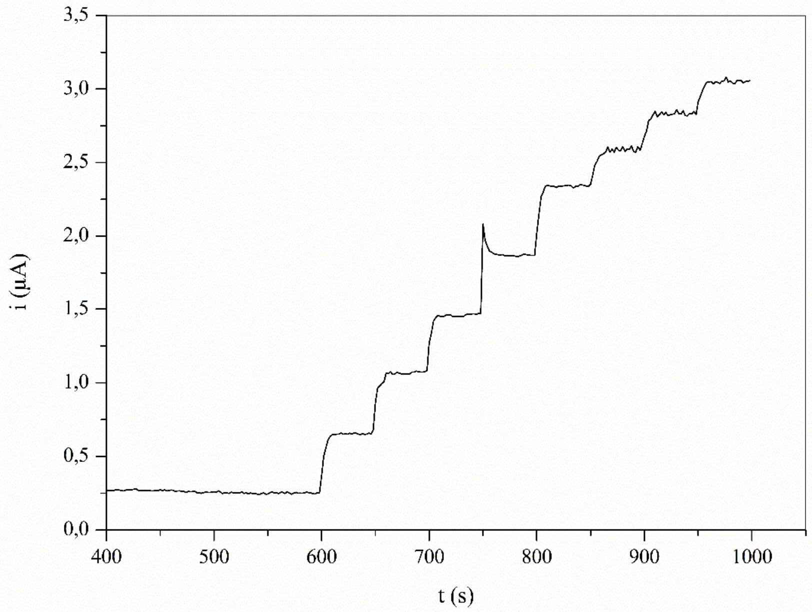

Figure S4: Amperogram for the measurement of saliva sample after the use of oral hygiene product. Electrochemical cell contained 20 mL 0.1 M PB (pH 7.4); the measurement was carried out at 0.65 V. Five additions of 5 µM standard solution were added, followed by three additions of 50 µL of saliva.
